# Feasibility and Safety Profile of Non‐anesthesiologist‐administered Propofol During Colorectal Endoscopic Submucosal Dissection: A Single‐center Study of 454 Procedures

**DOI:** 10.1002/deo2.70435

**Published:** 2026-09-16

**Authors:** Yumi Kishi, Mayo Tanabe, Naoyuki Uragami, Daijiro Shiomi, Mai Fukuda, Miho Kurahashi, Toshiki Hanzawa, Michimasa Yamaguchi, Mikio Muraoka, Hatsuka Nakamura, Rei Miyake, Yukiko Okada, Yoichi Toyoda, Akiko Toshimori, Takayoshi Ito, Noboru Yokoyama, Haruhiro Inoue

**Affiliations:** ^1^ Digestive Diseases Center Showa Medical University Koto Toyosu Hospital Tokyo Japan

**Keywords:** aged, colorectal neoplasms, endoscopic submucosal dissection, moderate sedation, propofol

## Abstract

**Objectives:**

Studies of non‐anesthesiologist‐administered propofol (NAAP) during colorectal endoscopic submucosal dissection (ESD) are scarce, particularly in the elderly. We evaluated its feasibility across a broad age range and identified factors associated with sedation‐related adverse events.

**Methods:**

We retrospectively analyzed 454 consecutive colorectal ESD procedures performed with NAAP in 405 patients at a tertiary center (January 2016–May 2024), stratified by age (<65, 65–74, and ≥75 years). Propofol was given by the endoscopy team as a weight‐based induction bolus and a continuous infusion titrated to target depth under multiparameter monitoring. New‐onset hypotension, bradycardia, and hypoxemia were compared among groups, with multivariable logistic regression for independent predictors.

**Results:**

Mean age was 68.2 years; 54.8% of procedures were performed in men. Adverse events were common but all transient: hypotension 14.1%, bradycardia 14.8%, hypoxemia 11.9%, without procedure discontinuation, intubation, anesthesiologist rescue, death, or permanent sequelae. A composite event (33.3%) rose with age (24.7%, 36.1%, and 39.1%; *p* = 0.018). In multivariable analysis, longer procedure duration, measured as propofol administration time, was independently associated with composite events (adjusted odds ratio [aOR] 1.05 per 10 min; *p* = 0.011) and bradycardia (aOR 1.07 per 10 min; *p* = 0.002). Age ≥75 years (vs. <65 years) was independently associated with composite events (aOR 1.85; *p* = 0.027) and hypoxemia (aOR 2.55; *p* = 0.021).

**Conclusions:**

Propofol‐based NAAP allowed colorectal ESD to be completed across all age groups without sedation‐related procedure termination or escalation of care. Older age and longer procedure duration were associated with selected sedation‐related adverse events, providing clinically relevant information for risk assessment during colorectal ESD.

**Trial Registration:**

N/A.

## 1 Introduction

Endoscopic submucosal dissection (ESD) enables en bloc resection of superficial gastrointestinal neoplasms, offering advantages in histopathological assessment and recurrence reduction [1, 2]. Because ESD is technically demanding and often prolonged, reliable sedation is critical for both procedural efficiency and patient safety [3].

Propofol is widely used for its rapid onset, predictable pharmacokinetics, and short recovery [4]; compared with midazolam, it yields superior sedation quality and shorter discharge times without a significant increase in adverse events [5, 6]. Non‐anesthesiologist‐administered propofol (NAAP) has gained wide acceptance in high‐throughput endoscopy units for its operational efficiency and independence from anesthesiology staffing [7, 8, 9, 10], with safe implementation requiring structured training and continuous physiologic monitoring per established guidelines [3, 11, 12, 13, 14].

The safety and efficacy of NAAP have been reported in esophageal and gastric ESD, including in elderly patients [15, 16, 17, 18, 19, 20, 21, 22, 23, 24, 25], but studies specifically evaluating NAAP during colorectal ESD remain scarce. Few prospective studies have evaluated sedation specifically during colorectal ESD, and those available examined dexmedetomidine rather than propofol [26, 27]. Colorectal ESD also presents distinct challenges, including limited scope handling, patient movement, and prolonged procedure times, while age‐related physiological changes and comorbidities may further affect outcomes. These factors have not been systematically examined in this setting.

We evaluated the feasibility and safety profile of NAAP during colorectal ESD across a wide age range, with particular attention to elderly patients, and examined clinical and procedural factors associated with sedation‐related adverse events.

## 2 Methods

### 2.1 Study Design and Setting

This retrospective study was conducted at the Digestive Diseases Center of Showa Medical University Koto Toyosu Hospital, a tertiary referral institution in Tokyo, Japan. An opt‐out consent procedure was implemented, allowing patients to request exclusion of their data from analysis. The study protocol was approved by the institutional review board (IRB approval No. 2024‐181‐A) and conformed to the principles of the Declaration of Helsinki. In Japan, propofol is not formally approved for sedation during gastrointestinal endoscopy; this off‐label use was approved by the Ethics Committee of Showa Medical University (approval No. 14001) and by the Advanced Medical Care Committee of Showa Medical University Koto‐Toyosu Hospital.

### 2.2 Study Population

**FIGURE 1:**
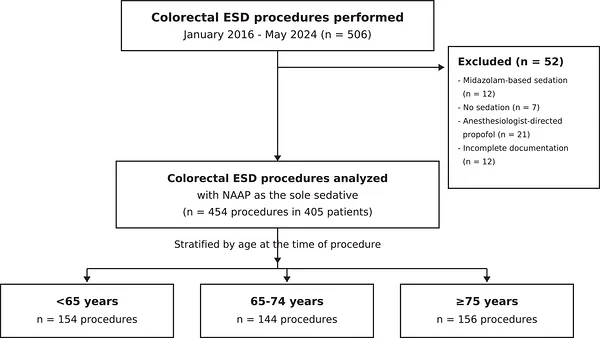
Flow diagram of procedure inclusion. Of 506 colorectal ESD procedures performed between January 2016 and May 2024, 52 were excluded, leaving 454 procedures in 405 patients for analysis. All included procedures were performed with NAAP as the sole sedative and were stratified according to patient age at the time of the procedure. ESD, endoscopic submucosal dissection; NAAP, non‐anesthesiologist‐administered propofol.

Patients who underwent colorectal ESD with NAAP between January 2016 and May 2024 were eligible. During this period, 506 colorectal ESD procedures were performed at our center. Fifty‐two cases were excluded for the following reasons: midazolam‐based sedation (*n* = 12), no sedation (*n* = 7), anesthesiologist‐directed propofol (*n* = 21), or incomplete sedation documentation (*n* = 12). The remaining 454 consecutive procedures, all performed with NAAP as the sole sedative and supported by complete medical records, constituted the final analytic cohort (Figure 1).

Patients were stratified into three age groups: <65 years, 65–74 years, and ≥75 years. Clinical, procedural, and sedation‐related variables were extracted from electronic medical records, including demographics, comorbidities, lesion characteristics, procedure time, and propofol dosing.

### 2.3 Sedation Protocol

Sedation was administered as NAAP under the supervision of an endoscopist credentialed in airway management, following current endoscopy guidelines. At our institution, gastroenterologists were authorized to administer propofol only after completing an institutional training program consisting of an in‐person or online lecture delivered by a board‐certified anesthesiologist and a post‐course assessment. The program was offered twice yearly and covered propofol pharmacology, patient selection, assessment of sedation depth, physiologic monitoring, airway management, and management of sedation‐related adverse events. Completion of both the lecture and the assessment was required for authorization; no minimum number of supervised cases was specified. The institutional NAAP program, including its training and authorization process, was approved and overseen by the hospital's Chief of Anesthesiology. Propofol was administered and monitored by an assisting physician and a registered nurse, independent of the endoscopist performing the procedure. Electrocardiography, pulse oximetry, respiratory rate, and capnography (end‐tidal CO_2_) were monitored continuously; noninvasive blood pressure and level of consciousness were assessed and recorded at 5‐min intervals. Sedation was initiated with propofol 0.5–1.5 mg/kg. The induction dose was selected by the treating physician according to the patient's age and general condition, with the lower end of the dosing range used for older or frail patients; no fixed age‐specific dosing algorithm was used. This was followed by a continuous infusion initiated at 3 mg/kg/h and titrated within a range of 2–6 mg/kg/h according to the clinical response to achieve a target Richmond Agitation‐Sedation Scale (RASS) score of −3, with temporary deepening to −4 when required for procedural stability [28]. Supplemental 10–20‐mg boluses were administered for inadequate sedation, and the reason for each dose adjustment or interruption was documented in the procedure record. Meperidine (pethidine) or pentazocine was administered as adjunctive analgesia at the discretion of the treating physician when additional analgesia was considered necessary. Fentanyl and benzodiazepines were not co‐administered. Hyoscine butylbromide (butylscopolamine) was used as an antiperistaltic agent unless contraindicated, and glucagon was used as an alternative when clinically indicated. The suite was equipped with supplemental oxygen, airway adjuncts, and resuscitative medications, and all sedation personnel had completed institutional training in managing sedation‐related complications.

### 2.4 Outcome Measures

The primary outcome was the incidence of sedation‐related adverse events during colorectal ESD: hypotension (systolic blood pressure <80 mmHg), bradycardia (heart rate <50/min), and hypoxemia (SpO_2_<90%). Each event was classified as new onset only when the corresponding baseline value did not meet the event threshold (systolic blood pressure ≥80 mmHg, heart rate ≥50/min, and SpO_2_ ≥90%, respectively). At baseline before sedation, no patient met any of the prespecified event thresholds (systolic blood pressure <80 mmHg, heart rate <50/min, or SpO_2_<90%), and no patient was receiving supplemental oxygen. Vital signs were recorded at 5‐min intervals, and an event was counted if any single value met the threshold, regardless of duration. A composite event was defined as the occurrence of any one of the three; each component was also analyzed separately.

Secondary outcomes included propofol dosing, sedation interruptions and their triggers, histopathology, and procedure‐related complications (bleeding, perforation, post‐ESD coagulation syndrome). Procedure duration was defined as the propofol administration time, that is, the interval during which the patient received propofol sedation for the ESD procedure. Post‐ESD coagulation syndrome was defined as localized abdominal pain or tenderness corresponding to the ESD site, accompanied by fever or laboratory evidence of inflammation, without evidence of perforation.

### 2.5 Statistical Analysis

Continuous variables were assessed for normality using the Shapiro–Wilk test and compared among the three age groups using one‐way analysis of variance (ANOVA) or the Kruskal–Wallis test, as appropriate. Categorical variables were compared using the χ^2^ test or Fisher's exact test, as appropriate. To evaluate factors associated with each outcome, univariable and multivariable logistic regression models were fitted for four outcomes: composite sedation‐related adverse events, hypotension, bradycardia, and hypoxemia. The following covariates were included: age group (<65, 65–74, and ≥75 years; <65 years as the reference), male sex, body mass index (BMI) ≥25 kg/m^2^, American Society of Anesthesiologists (ASA) class ≥III, and procedure duration (per 10‐min increase). Because some patients underwent multiple procedures (454 procedures in 405 patients), multivariable models were estimated using patient‐clustered robust standard errors. As a sensitivity analysis, all models were refitted using only the first eligible procedure for each patient (*n* = 405). Crude and adjusted odds ratios (cORs and aORs) with 95% confidence intervals (CIs) were calculated. As an exploratory analysis, adverse‐event rates were compared above and below a 90‐min threshold, a clinically practical cutoff for prolonged procedures. In sensitivity analyses, the composite model was refitted after separately adding cardiovascular disease and hypertension (Table S1) and adjunctive meperidine use (Table S2). Because total propofol dose was closely related to propofol administration time and dosing was titrated according to clinical response, propofol‐related variables were not included in the primary model. A two‐sided *p*‐value <0.05 was considered significant. Conventional statistical analyses were performed using JMP 17.0 (SAS Institute, Cary, NC, USA), whereas patient‐clustered regression and first‐procedure sensitivity analyses were performed using R version 4.6.1 (R Foundation for Statistical Computing, Vienna, Austria).

## 3 Results

### 3.1 Patient Characteristics

The 454 procedures were performed in 405 patients. Mean age at the time of the procedure was 68.2 ± 12.2 years, 54.8% of procedures were performed in men, and median procedure duration was 68 min (interquartile range 42–101). Baseline characteristics are shown in Table 1. BMI and several comorbidities differed across age groups, whereas procedure duration did not (*p* = 0.661).

**TABLE 1:** Baseline patient and procedural characteristics by age group.

Parameter	<65 years	65–74 years	≥75 years	*p*‐Value
*N*	154	144	156	
Age (years)	54.2 ± 7.8	70.2 ± 2.8	80.3 ± 4.2	<0.001
Male, *n* (%)	88 (57.1)	91 (63.2)	70 (44.9)	0.005
BMI (kg/m^2^)	23.7 ± 3.5	24.0 ± 4.9	22.7 ± 3.9	0.036
Lesion diameter (mm)	27.3 ± 13.4	26.5 ± 13.6	27.0 ± 15.1	0.850
Procedure duration (min), median [IQR]	64 [41–96]	71 [39–105]	69 [44–95]	0.661
**Lesion location, *n* (%)**				
Cecum	18 (11.7)	21 (14.6)	22 (14.1)	0.731
Ascending colon	38 (24.7)	43 (29.9)	34 (21.8)	0.268
Transverse colon	31 (20.1)	33 (22.9)	42 (26.9)	0.364
Descending colon	9 (5.8)	7 (4.9)	5 (3.2)	0.535
Sigmoid colon	26 (16.9)	19 (13.2)	25 (16.0)	0.656
Rectum	32 (20.8)	21 (14.6)	28 (17.9)	0.294
**Comorbidity, *n* (%)**				
Hypertension	37 (24.0)	72 (50.0)	84 (53.8)	<0.001
Diabetes mellitus	7 (4.5)	25 (17.4)	29 (18.6)	<0.001
Cardiovascular disease	5 (3.2)	20 (13.9)	45 (28.8)	<0.001
Pulmonary disease	5 (3.2)	8 (5.6)	11 (7.1)	0.321
Chronic kidney disease	2 (1.3)	5 (3.5)	5 (3.2)	0.437
Chronic liver disease	4 (2.6)	7 (4.9)	5 (3.2)	0.542
**ASA class, *n* (%)**				0.0003
I	61 (39.6)	35 (24.3)	37 (23.7)	
II	91 (59.1)	103 (71.5)	104 (66.7)	
III	2 (1.3)	6 (4.2)	15 (9.6)	
IV	0 (0.0)	0 (0.0)	0 (0.0)	
**Antithrombotic therapy, *n* (%)**	5 (3.2)	19 (13.2)	39 (25.0)	<0.001
Antiplatelet drugs	3 (1.9)	7 (4.9)	17 (10.9)	
Vitamin K antagonist	0 (0.0)	2 (1.4)	3 (1.9)	
DOACs	2 (1.3)	7 (4.9)	15 (9.6)	
Others	0 (0.0)	3 (2.1)	4 (2.6)	

Data are mean ± SD, median [interquartile range, IQR], or *n* (%). ASA, American Society of Anesthesiologists; BMI, body mass index; DOACs, direct oral anticoagulants. *p*‐Values were calculated with one‐way ANOVA for normally distributed continuous variables, the Kruskal–Wallis test for non‐parametric continuous variables, and the χ^2^ test (or Fisher's exact test when expected cell counts <5) for categorical variables.

### 3.2 Propofol Dosing and Adjunctive Medications

Propofol dosing parameters are shown in Table 2. Induction doses, maintenance infusion rates, and total doses all declined with age (all *p* < 0.0001), whereas the weight‐adjusted maintenance dose was similar across groups (*p* = 0.764; Table 2). Glucagon use increased with age (*p* = 0.003), while use of hyoscine butylbromide, meperidine, and pentazocine did not differ significantly.

**TABLE 2:** Propofol dosing metrics and adjunctive medications during non‐anesthesiologist‐administered propofol (NAAP)‐sedated colorectal endoscopic submucosal dissection (ESD).

Parameter	<65 years	65–74 years	≥75 years	*p*‐Value
Induction bolus (mg)	63.1 ± 20.3	55.8 ± 15.0	54.5 ± 18.4	<0.0001
Induction bolus (mg/kg)	1.00 ± 0.37	0.92 ± 0.27	1.02 ± 0.39	0.039
Maintenance dose (mg/h)	194.9 ± 47.3	182.0 ± 40.1	160.0 ± 38.8	<0.0001
Maintenance dose (mg/kg/h)	3.01 ± 0.55	2.99 ± 0.54	2.96 ± 0.56	0.764
Total propofol dose (mg)	469.1 ± 254.4	425.2 ± 238.3	372.3 ± 239.7	<0.0001
Hyoscine butylbromide, *n* (%)	127 (82.5)	105 (72.9)	121 (77.6)	0.139
Glucagon, *n* (%)	17 (11.0)	23 (16.0)	40 (25.6)	0.003
Meperidine, *n* (%)	118 (76.6)	100 (69.4)	114 (73.1)	0.377
Pentazocine, *n* (%)	4 (2.6)	6 (4.2)	3 (1.9)	0.503

Data are mean ± SD or *n* (%). ESD, endoscopic submucosal dissection; NAAP, non‐anesthesiologist‐administered propofol. *p*‐Values were obtained with one‐way ANOVA when data were normally distributed and the Kruskal–Wallis test otherwise; categorical variables were compared using the χ^2^ test or Fisher's exact test.

### 3.3 Procedural and Pathological Outcomes

Procedural and pathological findings are summarized in Tables 2 and 3. Lesion size (*p* = 0.850) and overall pathological distribution (*p* = 0.101) did not differ among groups. Procedural complications were similar across groups: post‐ESD bleeding in four procedures (0.9%) and perforation in one (0.2%), with no aspiration pneumonia.

**TABLE 3:** Histopathological outcomes and endoscopic submucosal dissection (ESD)‐related complications.

Parameter	<65 years	65–74 years	≥75 years
**Pathology, *n* (%)**			
Sessile serrated lesion	26 (16.9)	21 (14.6)	11 (7.1)
Tubular adenoma (low grade)	33 (21.4)	28 (19.4)	38 (24.4)
Tubular adenoma (high grade)	33 (21.4)	34 (23.6)	24 (15.4)
Adenocarcinoma (M)	44 (28.6)	44 (30.6)	60 (38.5)
Adenocarcinoma (SM1)	5 (3.2)	8 (5.6)	9 (5.8)
Adenocarcinoma (SM2)	9 (5.8)	6 (4.2)	11 (7.1)
Adenocarcinoma (MP)	0 (0.0)	0 (0.0)	2 (1.3)
Neuroendocrine tumor	0 (0.0)	2 (1.4)	0 (0.0)
Others	4 (2.6)	1 (0.7)	1 (0.6)
**Procedural complications, *n* (%)**			
Muscle injury during ESD	17 (11.0)	11 (7.6)	13 (8.3)
Post‐ESD coagulation syndrome	6 (3.9)	4 (2.8)	3 (1.9)
Delayed perforation	1 (0.6)	0 (0.0)	0 (0.0)
Delayed bleeding	0 (0.0)	2 (1.4)	2 (1.3)

Values are *n* (%).

Abbreviations: M, intramucosal cancer; MP, muscularis propria invasion; SM1, submucosal invasion <1000 µm; SM2, submucosal invasion ≥1000 µm.

### 3.4 Sedation‐related Adverse Events

New‐onset hypotension occurred in 64 procedures (14.1%), bradycardia in 67 (14.8%), and hypoxemia in 54 (11.9%); 151 procedures (33.3%) had at least one event (Table 4). Hypoxemia (6.5%, 11.8%, and 17.3%; *p* = 0.013) and the composite event (24.7%, 36.1%, and 39.1%; *p* = 0.018) increased with age, whereas hypotension (*p* = 0.293) and bradycardia (*p* = 0.382) did not. All documented events resolved with standard bedside measures (transient propofol dose reduction, verbal stimulation, jaw thrust, supplemental oxygen, or fluid loading), and no procedure was discontinued for a sedation‐related reason.

**TABLE 4:** Sedation‐related adverse events and causes of temporary propofol interruption.

Parameter	<65 years	65–74 years	≥75 years	*p*‐Value
Composite sedation‐related AE	38 (24.7)	52 (36.1)	61 (39.1)	0.018
Hypotension (SBP <80 mmHg)	17 (11.0)	25 (17.4)	22 (14.1)	0.293
Bradycardia (HR <50/min)	20 (13.0)	19 (13.2)	28 (17.9)	0.382
Hypoxemia (SpO2 <90%)	10 (6.5)	17 (11.8)	27 (17.3)	0.013
Procedure discontinued due to sedation	0 (0.0)	0 (0.0)	0 (0.0)	—
Temporary propofol interruption	13 (8.4)	12 (8.3)	17 (10.9)	0.681
Restlessness/body movement	4 (2.6)	3 (2.1)	1 (0.6)	0.399
Respiratory fluctuation	5 (3.)	6 (4.2)	8 (5.1)	0.710
Hemodynamic change	4 (2.6)	3 (2.1)	8 (5.1)	0.281

Values are n (%). A composite sedation‐related adverse event was defined as the occurrence of any one of hypotension, bradycardia, or hypoxemia. SBP, systolic blood pressure; HR, heart rate. *p*‐Values were calculated with the χ^2^ test or Fisher's exact test. “—” indicates that statistical testing was not applicable because all values were zero.

Temporary propofol interruption occurred in 42 procedures (9.3%) without inter‐group difference (8.4%, 8.3%, and 10.9%; *p* = 0.681), triggered by respiratory fluctuation (*n* = 19), hemodynamic change (*n* = 15), or restlessness (*n* = 8). No sedation was permanently discontinued, and no procedure required endotracheal intubation, conversion to general anesthesia, or anesthesiologist rescue. There were no sedation‐related deaths and no events resulting in permanent sequelae.

### 3.5 Independent Predictors of Sedation‐related Adverse Events

In multivariable models with patient‐clustered robust standard errors (Table 5), procedure duration was associated with the composite event (aOR 1.05 per 10 min, 95% CI 1.01–1.09; *p* = 0.011), as was age ≥75 years versus <65 years (aOR 1.85, 95% CI 1.07–3.19; *p* = 0.027). The association for age 65–74 years did not reach statistical significance (aOR 1.66, 95% CI 0.96–2.87; *p* = 0.069).

**TABLE 5:** Univariable and multivariable logistic regression for sedation‐related adverse events.

Variable	cOR (95% CI)	*p*‐Value	aOR (95% CI)	*p*‐Value
**Composite sedation‐related adverse event (*n* = 151/454)**				
65–74 years (vs. <65)	1.73 (1.05–2.84)	0.032	1.66 (0.96–2.87)	0.069
≥75 years (vs. <65)	1.96 (1.20–3.19)	0.007	1.85 (1.07–3.19)	**0.027**
Male sex	0.82 (0.56–1.22)	0.335	0.83 (0.53–1.30)	0.414
BMI ≥25 kg/m^2^	0.86 (0.55–1.35)	0.514	0.92 (0.57–1.48)	0.730
ASA class ≥III	1.58 (0.68–3.70)	0.289	1.44 (0.56–3.74)	0.448
Procedure duration (per 10 min)	1.05 (1.02–1.09)	0.001	1.05 (1.01–1.09)	**0.011**
**Hypoxemia (SpO_2_ <90%) (n = 54/454)**				
65–74 years (vs. <65)	1.93 (0.85–4.36)	0.115	1.93 (0.85–4.38)	0.116
≥75 years (vs. <65)	3.01 (1.40–6.47)	0.005	2.55 (1.15–5.63)	**0.021**
Male sex	0.68 (0.38–1.20)	0.181	0.63 (0.34–1.16)	0.140
BMI ≥25 kg/m^2^	1.40 (0.76–2.58)	0.273	1.46 (0.78–2.75)	0.236
ASA class ≥III	2.82 (1.06–7.49)	0.038	2.41 (0.85–6.83)	0.097
Procedure duration (per 10 min)	1.02 (0.98–1.07)	0.259	1.02 (0.98–1.06)	0.401
**Hypotension (SBP <80 mmHg) (*n* = 64/454)**				
65–74 years (vs. <65)	1.69 (0.87–3.29)	0.120	1.63 (0.82–3.26)	0.164
≥75 years (vs. <65)	1.32 (0.67–2.60)	0.417	1.38 (0.71–2.67)	0.341
Male sex	1.07 (0.63–1.82)	0.808	1.02 (0.59–1.77)	0.942
BMI ≥25 kg/m^2^	1.16 (0.65–2.08)	0.615	1.21 (0.66–2.22)	0.529
ASA class ≥III	0.91 (0.26–3.15)	0.882	0.85 (0.23–3.17)	0.811
Procedure duration (per 10 min)	1.00 (0.96–1.05)	0.892	1.00 (0.97–1.04)	0.828
**Bradycardia (HR <50/min) (*n* = 67/454)**				
65–74 years (vs. <65)	1.02 (0.52–2.00)	0.958	0.94 (0.46–1.93)	0.872
≥75 years (vs. <65)	1.47 (0.79–2.73)	0.229	1.53 (0.76–3.10)	0.233
Male sex	1.17 (0.69–1.98)	0.549	1.44 (0.79–2.62)	0.233
BMI ≥25 kg/m^2^	0.48 (0.24–0.95)	0.036	0.49 (0.24–0.99)	**0.046**
ASA class ≥III	0.86 (0.25–2.98)	0.812	0.72 (0.18–2.92)	0.645
Procedure duration (per 10 min)	1.06 (1.03–1.10)	0.001	1.07 (1.02–1.11)	**0.002**

Abbreviations: aOR, adjusted odds ratio; ASA, American Society of Anesthesiologists; BMI, body mass index; CI, confidence interval; cOR, crude odds ratio; HR, heart rate; SBP, systolic blood pressure. Multivariable models simultaneously included age group, male sex, BMI ≥25 kg/m^2^, ASA class ≥III, and procedure duration as covariates, and were estimated with robust standard errors clustered by patient (454 procedures in 405 patients). All univariable and multivariable models were fitted using all 454 procedures. Bold entries denote variables with *p* < 0.05 in the multivariable analysis.

Among individual events, procedure duration was associated with bradycardia (aOR 1.07 per 10 min, 95% CI 1.02–1.11; *p* = 0.002), whereas BMI ≥25 kg/m^2^ was inversely associated (aOR 0.49, 95% CI 0.24–0.99; *p* = 0.046). Age ≥75 years was associated with hypoxemia (aOR 2.55, 95% CI 1.15–5.63; *p* = 0.021); no covariate was independently associated with hypotension.

The associations of age and procedure duration were materially unchanged after adding cardiovascular disease and hypertension (Table S1), adding meperidine use (Table S2), or restricting the analysis to the first procedure per patient. In the first‐procedure analysis, the corresponding estimates remained significant for age ≥75 years (aOR 1.77, 95% CI 1.04–3.00; *p* = 0.034) and procedure duration (aOR 1.06 per 10 min, 95% CI 1.02–1.09; *p* = 0.002).

Procedures lasting ≥90 min had higher rates of composite events (44.4% vs. 28.1%; *p* = 0.001) and bradycardia (22.9% vs. 11.0%; *p* = 0.002), whereas hypotension, hypoxemia, and temporary propofol interruption did not differ significantly (Table 6).

**TABLE 6:** Sedation‐related adverse events stratified by procedure duration (<90 vs. ≥90 min).

Adverse event	<90 min (*n* = 310)	≥90 min (*n* = 144)	*p*‐Value
Composite adverse event	87 (28.1)	64 (44.4)	**0.001**
Hypotension (SBP <80 mmHg)	42 (13.5)	22 (15.3)	0.664
Bradycardia (HR <50/min)	34 (11.0)	33 (22.9)	**0.002**
Hypoxemia (SpO_2_ <90%)	32 (10.3)	22 (15.3)	0.160
Temporary propofol interruption	29 (9.4)	13 (9.0)	1.000

Values are *n* (%). A composite adverse event was defined as the occurrence of any one of hypotension, bradycardia, or hypoxemia. The 90‐min threshold was chosen to translate the continuous duration effect identified in multivariable analysis into a clinically practical threshold. SBP, systolic blood pressure; HR, heart rate. *p*‐Values were calculated using Fisher's exact test.

## 4 Discussion

To our knowledge, this is the first study to specifically evaluate non‐anesthesiologist‐administered propofol during colorectal ESD. While NAAP has been studied in esophageal and gastric ESD and in colonoscopy [9, 10, 15, 18], sedation for colorectal ESD has not been standardized, and the few prospective studies in this setting evaluated dexmedetomidine rather than propofol [26, 27]. The present data therefore address a gap in the evidence for a widely used ESD sedation strategy.

Each individual adverse event occurred in about 12%–15% of procedures. Our event definition was intentionally sensitive, counting any threshold‐crossing value regardless of duration, so brief self‐limited deviations were also captured; nevertheless, the observed rates were within previously reported ranges for propofol [9, 22]. We therefore interpret the findings in terms of feasibility and manageability rather than absolute safety.

Within these limits, these findings suggest that NAAP is feasible during colorectal ESD across a wide age range, when delivered within a structured institutional framework. Reproducibility depends on several components of our protocol: oversight by the Chief of Anesthesiology; an anesthesiologist‐led training program offered twice yearly, covering propofol pharmacology, patient selection, assessment of sedation depth, physiologic monitoring, airway management, and management of sedation‐related adverse events, followed by a post‐course assessment; institutional authorization of participating gastroenterologists; sedation personnel independent of the procedural endoscopist; continuous multiparameter monitoring; and immediate availability of airway and resuscitation equipment. The target depth of moderate‐to‐deep sedation (RASS −3 to −4) should also be considered when interpreting and replicating the protocol. Accordingly, these findings may not be generalizable to settings without comparable training, staffing, monitoring, and rescue capability, and implementation should be adapted to local regulations and resources.

Absolute propofol doses decreased with age, whereas weight‐adjusted maintenance doses were comparable across age groups, suggesting that body weight contributed substantially to the observed difference. Each 10‐min increase in procedure duration was associated with 5% higher odds of a composite event and 7% higher odds of bradycardia. Because procedure duration is partly determined by lesion complexity and technical difficulty, causality cannot be inferred; the association may reflect greater procedural complexity and cumulative sedative exposure rather than elapsed time itself, and residual confounding from unmeasured factors not included in the model, such as fibrosis. Older age was independently associated with composite events and hypoxemia, and the duration effect persisted after adding cardiovascular disease and hypertension (Table S1) or adjunctive meperidine (Table S2). The inverse association between BMI ≥25 kg/m^2^ and bradycardia is difficult to explain and may be a chance finding. Bradycardia during colorectal procedures is likely multifactorial: beyond propofol, luminal insufflation and bowel distension can elicit vagally mediated reflex bradycardia, so the observed events may not be attributable to sedation alone. Because insufflation volume and vagal responses were not recorded, this contribution could not be quantified.

These findings should be interpreted in the context of current guidelines. ASGE and ESGE/ESGENA guidelines support propofol sedation by appropriately trained non‐anesthesia personnel within suitable staffing, monitoring, and legal frameworks [11, 12]. The JGES guideline permits propofol administration by physicians trained in airway management in ASA‐PS I–II patients and recommends anesthesiologist consultation when determining sedation for patients with ASA‐PS III or higher; it also notes the absence of a standardized national NAAP education system [3]. In this cohort, 94.9% of procedures involved ASA‐PS I–II patients and 5.1% involved ASA‐PS III patients; the latter subgroup should be interpreted cautiously.

Evidence comparing propofol with midazolam is mixed. Some studies report a higher hypotension risk with propofol [15], whereas meta‐analyses show no difference in major cardiovascular outcomes [6, 18, 25], and propofol offers faster recovery and discharge than traditional sedatives [17, 22, 24, 29]. Remimazolam has emerged as a reversible alternative with a favorable cardiopulmonary profile [30, 31]. Propofol may be a practical option for prolonged colorectal ESD because it can be continuously infused and rapidly titrated; however, its comparative advantages over alternative sedatives remain uncertain.

This study has several limitations. First, its retrospective single‐center design may limit generalizability. Second, some patients underwent more than one procedure during the study period; although models used patient‐clustered robust standard errors and analyses restricted to first procedures yielded similar estimates, residual within‐patient correlation cannot be fully excluded. Third, adjunctive analgesics were not included in the primary models; meperidine use was similar across age groups and did not materially alter the results in sensitivity analyses (Table S2), but residual confounding cannot be excluded. Fourth, bispectral‐index monitoring was not used, and sedation depth was assessed clinically. Finally, the absence of a comparator arm prevents direct comparison with anesthesiologist‐directed or alternative‐agent sedation; during the study period, anesthesiologist‐directed sedation was used only during live endoscopy demonstrations, for operational rather than patient‐risk reasons.

In this cohort, propofol‐based NAAP allowed colorectal ESD to be completed across all age groups without sedation‐related procedure termination or escalation of care. Age ≥75 years and longer procedure duration were associated with selected sedation‐related adverse events. Because no comparator arm was included, these findings support feasibility but do not establish comparative safety. Prospective multicenter studies are warranted.

## Author Contributions

Yumi Kishi and Mayo Tanabe designed the study and drafted the manuscript. Naoyuki Uragami developed and maintained the study database. Daijiro Shiomi, Mai Fukuda, Miho Kurahashi, Toshiki Hanzawa, Michimasa Yamaguchi, Mikio Muraoka, Hatsuka Nakamura, Rei Miyake, Yukiko Okada, Yoichi Toyoda, and Akiko Toshimori collected the data. Yumi Kishi and Mayo Tanabe performed the statistical analysis. Naoyuki Uragami, Takayoshi Ito, Noboru Yokoyama, and Haruhiro Inoue critically revised the manuscript. All authors approved the final version.

## Funding

The authors have nothing to report.

## Ethics Statement

**Approval of the research protocol by an Institutional Review Board**: The study protocol was approved by the Institutional Review Board of Showa Medical University Koto Toyosu Hospital (approval No. 2024‐181‐A). The off‐label use of propofol was additionally approved by the Ethics Committee of Showa Medical University (approval No. 14001) and by the Advanced Medical Care Committee of Showa Medical University Koto Toyosu Hospital.

## Consent

Owing to the retrospective design, written informed consent was waived, and an opt‐out consent procedure was implemented, allowing patients to decline inclusion of their data.

## Conflicts of Interest

Haruhiro Inoue is an advisor for Olympus Corporation and Top Corporation, and has received educational grants from Olympus Corporation, Boston Scientific, and Takeda Pharmaceutical Co. The remaining authors declare no conflicts of interest.

## Supporting information




**Table S1** Sensitivity analysis for the composite sedation‐related adverse event, adding cardiovascular disease and hypertension to the primary multivariable model.
**Table S2** Sensitivity analysis for the composite sedation‐related adverse event, adding adjunctive meperidine use to the primary multivariable model.

## Data Availability

The data that support the findings of this study are available from the corresponding author upon reasonable request.
