# Supplementary material for: Feasibility and Safety Profile of Non‐anesthesiologist‐administered Propofol During Colorectal Endoscopic Submucosal Dissection: A Single‐center Study of 454 Procedures
Source: DEN Open. 2026 Sep 16;7(1):e70435. doi: 10.1002/deo2.70435 (PMC13582286; doi:10.1002/deo2.70435)
Supplement: Supplementary file 1 [file DEO2-7-e70435-s001.docx]

**Table S1. Sensitivity analysis for the composite adverse event with additional comorbidity covariates**

| **Variable** | **cOR (95% CI)** | **P value** | **aOR (95% CI)** | **P value** |
| --- | --- | --- | --- | --- |
| 65–74 years (vs <65) | 1.73 (1.05–2.84) | 0.032 | 1.84 (1.07–3.19) | **0.028** |
| ≥75 years (vs <65) | 1.96 (1.20–3.19) | 0.007 | 2.21 (1.24–3.95) | **0.007** |
| Male sex | 0.82 (0.56–1.22) | 0.335 | 0.89 (0.57–1.41) | 0.625 |
| BMI ≥25 kg/m² | 0.86 (0.55–1.35) | 0.514 | 0.97 (0.60–1.58) | 0.906 |
| ASA class ≥III | 1.58 (0.68–3.70) | 0.289 | 1.82 (0.67–4.95) | 0.239 |
| Procedure duration (per 10-min) | 1.05 (1.02–1.09) | 0.001 | 1.05 (1.01–1.09) | **0.011** |
| Cardiovascular disease | 0.84 (0.48–1.46) | 0.529 | 0.68 (0.34–1.38) | 0.283 |
| Hypertension | 0.84 (0.57–1.25) | 0.399 | 0.75 (0.46–1.21) | 0.239 |

*Sensitivity analysis for composite events (151/454), adding cardiovascular disease and hypertension to the primary model. Adjusted estimates used patient-clustered robust standard errors. cOR, crude odds ratio; aOR, adjusted odds ratio; CI, confidence interval; BMI, body mass index; ASA, American Society of Anesthesiologists. Bold indicates P<0.05 in the multivariable analysis.*

**Table S2. Sensitivity analysis for the composite adverse event with adjunctive meperidine use**

| **Variable** | **cOR (95% CI)** | **P value** | **aOR (95% CI)** | **P value** |
| --- | --- | --- | --- | --- |
| 65–74 years (vs <65) | 1.73 (1.05–2.84) | 0.032 | 1.71 (0.99–2.96) | 0.056 |
| ≥75 years (vs <65) | 1.96 (1.20–3.19) | 0.007 | 1.86 (1.08–3.22) | **0.026** |
| Male sex | 0.82 (0.56–1.22) | 0.335 | 0.83 (0.53–1.32) | 0.435 |
| BMI ≥25 kg/m² | 0.86 (0.55–1.35) | 0.514 | 0.90 (0.56–1.45) | 0.678 |
| ASA class ≥III | 1.58 (0.68–3.70) | 0.289 | 1.64 (0.64–4.23) | 0.302 |
| Procedure duration (per 10-min) | 1.05 (1.02–1.09) | 0.001 | 1.06 (1.02–1.10) | **0.007** |
| Meperidine use | 1.33 (0.85–2.10) | 0.211 | 1.56 (0.97–2.50) | 0.064 |

*Sensitivity analysis for the composite sedation-related adverse event (n=151/454), adding adjunctive meperidine use to the primary multivariable model.* *Adjusted estimates used patient-clustered robust standard errors. cOR, crude odds ratio; aOR, adjusted odds ratio; CI, confidence interval; BMI, body mass index; ASA, American Society of Anesthesiologists. Bold entries denote P<0.05 in the multivariable analysis.*
